# Fluorescent In Situ Hybridization Testing Allows the Diagnosis of *NRG1* Gene Fusions in Lung and Pancreas Cancers with No Other Identified Oncogenic Driver

**DOI:** 10.3390/cancers17142347

**Published:** 2025-07-15

**Authors:** Clara Bastard, Charline Caumont, Laura Samaison, Isabelle Quintin-Roué, Laurent Doucet, Pascale Marcorelles, Cédric Le Maréchal, Jean-Philippe Merlio, David Cappellen, Arnaud Uguen

**Affiliations:** 1 CHU Brest, Service d’Anatomie et Cytologie Pathologiques, F-29200 Brest, France; 2 CHU Bordeaux, Department of Tumor Biology, F-33600 Pessac, Francedavid.cappellen@u-bordeaux.fr (D.C.); 3 BRIC (BoRdeaux Institute of onCology), UMR1312, INSERM, Univ. Bordeaux, F-33000 Bordeaux, France; 4 Tumor Bank and Tumor Biology Laboratory, Bordeaux University Hospital, F-33600 Pessac, France; 5 Ouest Pathologie, F-29000 Quimper, France; 6 CHU Brest, Service de Génétique Moléculaire, F-29200 Brest, France; 7 Univ Brest, Inserm, CHU de Brest, UMR1078, F-29200 Brest, France; 8 Univ Brest, Inserm, CHU de Brest, LBAI, UMR1227, F-29200 Brest, France

**Keywords:** *NRG1* fusion, pancreatic cancer, lung cancer, fluorescent in situ hybridization, RNA-sequencing

## Abstract

Some pancreatic ductal-type (PDADK) and lung adenocarcinomas (LADK) lacking other molecular drivers are reported to harbor *NRG1* fusions as potential novel therapeutic targets. We investigated the feasibility of a fluorescent in situ hybridization (FISH)-based diagnosis of *NRG1* fusions in a case series of PDADK and LADK lacking other identified oncogenic drivers. First, among a case series of PDADK, *KRAS* analyses (PCR followed in PCR-negative cases by RNA sequencing—RNAseq) found 27/162 (16.7%) *KRAS* wild-type cases, among which 1/162 (0.6%) *NRG1* fusion was diagnosed using FISH. Secondly, among a case series of LDAK, 191/446 (42.8%) cases had no molecular alterations in *EGFR*, *KRAS*, *BRAF*, *HER2*, *MET*, *ALK*, *ROS1* and *RET* according to NGS and FISH analyses and, among them, 4/446 (0.9%) cases had *NRG1* fusions using FISH. Finally, four additional cases out of the two previously mentioned cases series (1 PDADK and 3 LADK) with *NRG1* fusions diagnosed by first-line RNAseq were also concluded as *NRG1* FISH-positive. The *NRG1* FISH tests for the nine *NRG1* FISH-positive cases resulted in 50% to 80% of positive tumor nuclei, all with single 3′-*NRG1* FISH signals. In our series, of the 22 cases analyzed with both *NRG1* FISH (positivity criterion of at least 15% of tumor nuclei with a split between the 5′- and the 3′- parts of the probes and/or isolated single 3′-*NRG1* signal) and RNAseq, 17 cases were FISH– RNAseq– and 5 cases were FISH+ RNAseq+ (no FISH+ RNAseq– or FISH– RNAseq+ cases in our study) resulting in 100% sensibility and specificity for the *NRG1* FISH test. In the case of no access to RNAseq, *NRG1* FISH consists of a valuable tool searching for *NRG1* fusions in patients with advanced cancers.

## Simple Summary

Pancreatic ductal carcinomas and lung adenocarcinomas are aggressive and deadly cancers, among which rare tumors harbor *NRG1* fusions making the *NRG1*-rearranged tumor candidates suitable for specific targeted therapies. Access to therapeutic innovation is conditioned by the diagnosis of *NRG1* fusion. In our study, we demonstrate the feasibility of an *NRG1* fusion screening using *NRG1* fluorescent in situ hybridization in pancreatic and lung tumors lacking other identified oncogenic drivers. In the case of no access to RNA sequencing, *NRG1* FISH consists of a valuable tool searching for *NRG1* fusions in patients with advanced cancers.

## 1. Introduction

Among the deadliest cancers are pancreatic ductal adenocarcinoma (PDADK) and non-small cell lung cancers (NSCLC), notably comprising an increasing proportion of lung adenocarcinomas (LADK). Being the fourth leading cause of cancer-related deaths, PDADK is projected to become the second leading cause of death in 2030. Meanwhile, NSCLC remains the leading cause of cancer mortality [1,2]. For PDADK, the only curative treatment is surgery at an early stage but, when diagnosed, only 20% of the patients have resectable tumors and, of those, only 20% survive for 5 years or longer. Most patients with PDADK are resistant to chemotherapy [3,4]. For LADK, about three out of four patients are diagnosed with advanced diseases and require systemic therapies from the first line of treatment. PD-1/PD-L1 immune checkpoint inhibition and targeted therapies against oncogenic drivers, such as EGFR, KRASG12C, MET, BRAFV600E, and HER2 mutants or ALK, ROS1, and RET chimeric proteins have improved the treatment of LADK, but the 5-year survival rate of patients remains low (26% for any stages, from 64% if diagnosed at local stages to 8% for diagnosis at metastatic stages) [5,6]. Thus, it remains crucial to investigate new therapeutic approaches to treat patients with PDADK and NSCLC.

The targeting of tumors with *Neuregulin 1* (*NRG1*) gene fusions could be one promising approach. Indeed, *NRG1* fusions drive cancer development through aberrant ERBB receptor-mediated signaling, and ERBB inhibition can inhibit the tumor growth of *NRG1*-rearranged cancers. Promising therapeutic responses have already been reported in patients with *NRG1*-rearranged PDADK and LADK [7,8,9,10,11,12]. *KRAS* wild-type PDADK were reported to frequently harbor *NRG1* fusions (until about 6% of PDADK in Jones et al.) and *NRG1*-rearranged *KRAS* wild-type PDADK seem particularly frequent in young patients (e.g., 4/17 23.5% of patients <50 years in Heining et al.) [7,8]. *NRG1* fusions are also reported in about 0.8% (0.3–2.7%) of patients with LADK, especially those without any known oncogenic mutations, young and non-smokers, and those with a particular subtype of mucinous LADK in which *NRG1* fusions are particularly frequent [13,14].

Screening for *NRG1* fusions is like searching for a needle in a haystack, and diagnostic strategies can vary from one cancer type to another and from one medical center/healthcare system to another because of various workflow, technical, and medico-economic reasons. RNA sequencing (RNAseq) is now the reference method for searching for gene fusions in cancers, but not every laboratory has implemented it in their routine practice thus far, even for tumors requiring the molecular analysis of numerous genes for therapeutic decisions, such as LADK [15]. Moreover, for some cancers, such as PDADK, molecular testing is not systematically performed, limiting the diagnosis of targetable molecular events, such as *NRG1* fusions [16,17].

The use of fluorescence in situ hybridization (FISH) to search for *NRG1* fusions has been reported in some studies, from reports on pan-cancer to selected histological subtypes, such as invasive mucinous adenocarcinoma of the lung, with the use of various probes (in-house-developed or commercial probes) [18,19,20]. Nevertheless, reports about *NRG1* FISH testing in real-life diagnosis and how it could be used as an alternative or complementary method in the era of RNAseq remain rare. Thus, in this study, we intend to evaluate the value of *NRG1* fusion diagnosis using FISH in a case series of PDADK and LADK, with a focus on cancers with no other identified molecular oncogenic driver.

## 2. Material and Methods

### 2.1. Cases Selection

The cases included in this study were (1) those with PDADK diagnosed in the CHRU Brest (France) between January 2000 and January 2020 for the PDADK case series of patients and (2) those who had a LADK tumor sample with molecular analyses performed for diagnostic purpose in CHRU Brest between January 2021 and June 2022 for the LADK case series of patients. In addition to these two exhaustive case series, 4 selected additional cases (1 PDADK and 3 LADK) with *NRG1* fusions diagnosed later (between 2023 and 2025) using first-line RNAseq were added to our study for method comparison purposes but were not counted in the calculation of *NRG1* fusion frequencies. Formalin-fixed paraffin-embedded (FFPE) tumor samples were collected from archives on the basis of initial pathology reports and, for PDADK, slides were digitalized to measure the sample surfaces and the percentages of tumor cells. For LADK, the quantification of tumor cells within the samples had been already performed during theranostic analyses. The present study was conducted in accordance with our national and institutional guidelines. All samples were included in a registered tumor tissue collection, and the present study was conducted in compliance with the Helsinki Declaration and approved by the Institutional Review Board of CHRU Brest (CPP n° AC-2019-3642—DC—2008—214, 11 August 2008).

### 2.2. Molecular Testing in PDADK

Based on 5 µm FFPE tumor sections, every PDADK of the cases series was first tested for *KRAS* mutations using the Idylla^TM^ KRAS mutation test (Biocartis, Mechelen, Belgium) to differentiate *KRAS*-mutated samples from *KRAS* wild-type samples. For *KRAS* wild-type samples, molecular analyses were pursued using RNAseq (Archer^®^ FusionPlex^®^ Lung panel, ArcherDX, Boulder, CO, USA, including *NRG1* in the panel of genes analyzed) and *NRG1* FISH (Zyto*Light*SPEC NRG1 Dual Color Break Apart, ZytoVision GmbH, Bremerhaven, Germany). The different analyses (i.e Idylla^TM^ KRAS testing, RNAseq and FISH ones) were performed following previously reported methodologies [17]. A threshold of at least 15% of tumor nuclei with a positive *NRG1* FISH pattern (i.e., a split between the 5′- and the 3′- parts of the probes and/or isolated single 3′-*NRG1* signal) was used to define a positive *NRG1* FISH result reflecting an *NRG1* fusion as used in previous studies [19,21].

### 2.3. Molecular Testing in LADK

Every LADK sample had initially been tested for *EGFR, KRAS, BRAF, HER2, MET* mutations and *ALK*, *ROS1*, *RET* fusions, as routinely performed in our daily diagnostic practice using non-RNAseq methods. *NRG1* fusion testing was performed on an additional tissue section using *NRG1* FISH in every sample, regardless of the molecular alteration that had been diagnosed or undiagnosed in the other genes. The same positivity criteria as those used for PDADK were used to define a positive *NRG1* FISH result reflecting an *NRG1* fusion.

### 2.4. Statistical Analyses

Descriptive statistical analyses, area under the receiver operating curve (AUROC) calculations and proportion comparisons (Chi-squared test) were performed using MedCalc Statistical Software version 13.2.2 (MedCalc Software bvba, Ostend, Belgium; 2014). The level of significance was set at *p* < 0.05. The specificity and sensibility of the *NRG1* FISH test were calculated using only the cases tested by both RNAseq and *NRG1* FISH, with RNAseq serving as the gold standard method.

## 3. Results

### 3.1. Cases Included

Samples from 199 patients with PDADK—112 men (56.6%) and 86 women (43.4%)—with a median age of 68.4 years (36 to 87 years) were included in this study. PDADK tumor samples were either surgical specimens (104, 52.5%) or biopsies (94, 47.5%). Samples between 2000 and 2009 (96, 48.5%) were fixed using acetic formalin, whereas those since 2010 were fixed using neutral buffered formalin 10% (102, 51.5%). Tissue surfaces ranged from 0.7 mm^2^ to 635.3 mm^2^ (mean surface area: 216.3 mm^2^ [95% I.C: 185.4–247.2]) with tumor cell contents of 1% to 100% (mean percentage: 31.3% [95% I.C: 28–34.6]), with 104 (52.5%) samples below the manufacturer’s recommendations for Idylla^TM^ KRAS mutation testing (i.e., at least 10% of tumor cells content in a tissue area between 50 and 600 mm^2^ for a 5 µm-thick tissue section).

For LADK, 446 patients were included—251 men (56.3%) and 195 women (43.7%)—with a median age of 66.9 years ranging from 36 to 91 years. LADK tumors samples were mostly (304, 68.2%) lung samples with 142 (31.8%) metastases at different sites (39 lymph nodes, 36 pleural, 20 brain, 12 bone, 12 liver, and 23 other visceral metastases). Every LADK sample was fixed using neutral buffered formalin 10%. Tumor cell contents ranged from 1% to 95% (mean 47.3% [95% I.C:44.7–49.8]) (tissue surfaces used for nucleic acid extraction were not measured for LADK in our routine practice). See Table 1 for the summarized data of patients and tumor samples.

### 3.2. Molecular Testing in PDADK

*KRAS* testing using the Idylla^TM^ KRAS Mutation Test resulted in contributive analyses in 162/199 (81.4%) samples and non contributive analyses in 37/199 (18.6%) samples. The number of invalid results within the samples fixed using acetic formalin from 2000 to 2009 was 37/96 (38.5%), whereas no invalid result was obtained in the 103 samples fixed using neutral buffered formalin (10%) since 2010 (*p* < 0.0001). Invalid results were obtained in 23/95 (24.2%) biopsy samples and in 14/104 (13.5%) surgical specimens (*p* = 0.0481). The valid/invalid results of the Idylla tests were also correlated to the tissue surface (AUROC 0.671, *p* = 0.0012) used for the analysis but not to the percentage of tumor cells within the tissue section (AUROC = 0.522, *p* = 0.661).

Among samples with contributive Idylla^TM^ *KRAS* analyses, a *KRAS* mutation was diagnosed in 130/162 (80.2%) cases (including 118 mutations in codon 12 and 12 mutations in codon 61) and no mutations were detected in 32/162 (19.7%) cases. RNAseq analyses in the 32 Idylla^TM^ *KRAS*-wild type samples reached contributive results in 18/32 (56.3%) samples and diagnosed 5 additional *KRAS* mutations (2 codon 12 and 3 codon 61 mutations) resulting in a final number of 27/162 (16.7%) *KRAS*-wild type PDADK samples. For both Idylla^TM^ and RNAseq, non contributive results were mostly obtained in samples fixed using acetic formalin and/or small biopsy samples. RNAseq analyses also detected a *BRAFD594G* mutation in one case and two gene fusions in two other cases: an *OR2T12-NTRK3* fusion and an *ATP1B1-NRG1* fusion.

The PDADK with the *ATP1B1-NRG1* fusion (Pancreas #1) had 80% *NRG1* FISH-positive nuclei with single 3′-*NRG1* signals (whereas among the PDADK samples with no *NRG1* fusion, *NRG1* FISH results did not exceed 2% of positive nuclei). Thus, the frequency of *NRG1*-rearranged PDADK was 0.6% (1/162) in our series. A PDADK case out of the series (Pancreas #2) and diagnosed using first-line RNAseq as having a *CDH1*-*NRG1* fusion was also *NRG1* FISH-positive with 60% FISH-positive nuclei with single 3′-*NRG1* signals. No split and no single 5′ signals were observed in Pancreas #1 and #2.

### 3.3. Molecular Testing in LADK

Among the 446 LADK samples, *EGFR* mutations had been diagnosed in 49 cases (11%), *KRAS* mutations in 159 cases (35.7%), *BRAF* mutations in 20 cases (4.5%), *HER2* mutations in 2 cases (0.4%), *MET* exon 14 skipping mutations in 13 cases (2.9%), *ALK* fusions in 12 cases (2.7%), *ROS1* fusions in 1 case (0.2%) and *RET* fusions in 4 cases (0.9%) (double mutations were encountered in five cases: four double *KRAS* and *BRAF* mutations and one case with *EGFR* and *BRAF* mutations). Moreover, 191 (42.8%) cases had no mutation/gene fusion detected in *EGFR, KRAS, BRAF, HER2, MET, ALK, ROS1* or *RET* genes.

*NRG1* FISH tests resulted in percentages of positive nuclei higher than the 15% threshold in four cases of the series (cases Lung #1 to Lung #4, between 50% and 80% of positive nuclei, all with single 3′-*NRG1* signals FISH patterns). The vast majority of FISH results were below the positivity threshold (mean 1% of positive nuclei in samples below the 15% threshold ranging from 0.5% to 5%). In this manner, the four cases, Lung #1 to Lung #4, were concluded as bearing *NRG1* fusions on the basis of the FISH test, resulting in an *NRG1* fusion frequency of 0.9% in our cases series of LADK. Unfortunately, insufficient tumor material provided additional confirmatory RNAseq analyses for these four cases.

Three additional LADK cases out of the series (Lung #5 to Lung #7), diagnosed using first-line RNAseq as having *NRG1* fusions (2 *CD74-NRG1* and 1 *ADAM9-NRG1* fusions), were also *NRG1* FISH-positive with 50% to 80% of FISH positive nuclei with single 3′-*NRG1* signals. No split and no single 5′ signals were observed in Lung #1 to #7.

### 3.4. Specificity and Sensitivity of NRG1 FISH Test

Based on the *NRG1* FISH results from 22 cases with contributive results using both RNAseq and FISH (19 PDADK and 3 LADK—comprising 17 FISH-negative/RNAseq-negative cases and 5 FISH-positive/RNAseq-positive cases), the *NRG1* FISH assay with a 15% positive-nuclei threshold achieved 100% sensitivity and 100% specificity for detecting tumors with *NRG1* fusions, with no false positives or false negatives.

**Figure 1:**
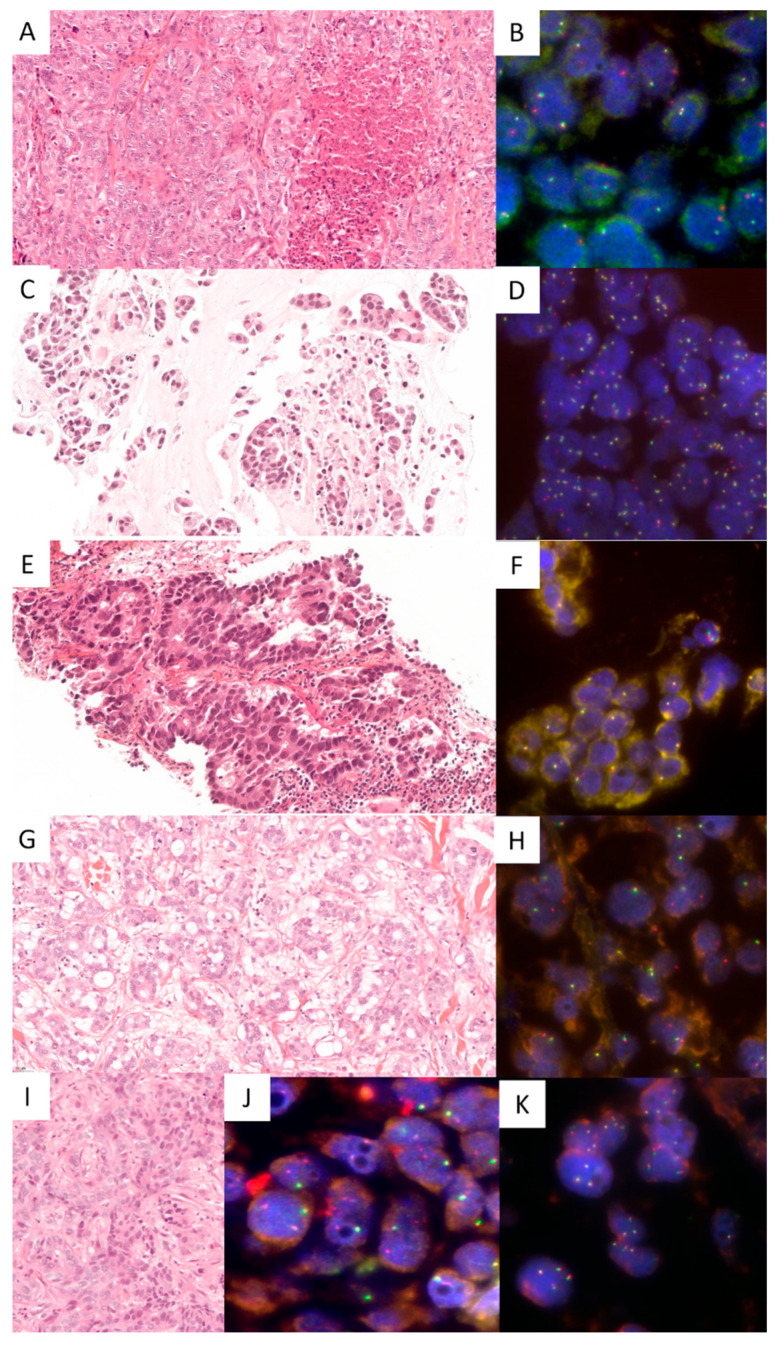
Histopathological and *NRG1* fluorescent in situ hybridization (FISH) images of tumors with *NRG1* fusions (Lung #1: (**A**,**B**); Lung #2: (**C**,**D**); Lung #3: (**E**,**F**); Lung #4: (**G**,**H**); Pancreas #1: (**I**,**J**); example of *NRG1* FISH-negative result in a RNAseq negative pancreatic tumor: (**K**); (**A**,**C**,**E**,**G**,**I**): hematoxylin eosin saffron, ×20 magnification; (**B**,**D**,**F**,**H**,**J**,**K**): Zyto*Light* SPEC NRG1 Dual Color Break Apart probe, DAPI counterstaining, ×100 magnification).

Summarized data about the cases with *NRG1* fusions appear in Table 2, and some cases are illustrated in Figure 1.

## 4. Discussion

Strategies to reach the diagnosis (and potentially the therapeutic targeting) of *NRG1* fusions can vary between PDADK and LADK.

According to the initial report of 6% *NRG1* fusion frequency in PDADK, we expected to diagnose about 12 *NRG1* fusions in our case series [8]. The fact that we detected only 1 (0.6%) *NRG1*-rearranged PDADK is ultimately more concordant with other publications reporting inferior frequencies of *NRG1* fusions in about 0.13–0.5% of PDADK, only with a possible overestimation of this frequency in the initial reports and/or also potential variations between different geographical areas and populations [22,23]. According to our results and literature data, to focus the testing for gene fusions, *NRG1* and others as *NTRK3*, on *KRAS*-wild type PDADK still makes sense. This suggests performing molecular testing in PDADK, either searching for *KRAS* mutations together with other molecular alterations including genes fusions using RNAseq or with first-line *KRAS*-focused testing (e.g., using Idylla^TM^ test) to identify the *KRAS*-wild type tumors meriting a complementary molecular testing.

RNAseq is particularly useful for analyzing LADK tumor samples given (1) the variety and frequency of targetable gene fusions in patients with LADK, (2), the ability of RNAseq methods to combine the detection of fusions and mutations, and (3) the small size of tumor samples to analyze, often consisting of biopsy specimens, limiting the multiplication of single-target analyses. Nevertheless, in case of no access to RNAseq for technical and economic reasons, easy-to-implement methods such as FISH tests are also logical for searching for gene fusions including *NRG1*, especially in cases with no other identified oncogenic driver. The 0.9% frequency of FISH-diagnosed *NRG1* fusions in our case series of LADK is consistent with the frequencies of *NRG1* fusions reported in NSCLC in the literature [13].

*NRG1* fusions can involve more than 150 different partners beyond the most common *CD74* gene (which accounts for more than 10% of *NRG1* fusions). Although an *NRG1* FISH test with probes targeting the *NRG1* locus cannot identify the fusion partner, it has the advantage of allowing the diagnosis of an *NRG1* fusion independent of the partner [24]. In our study, although not every LADK case allowed for confirmatory RNAseq analyses, the strong (100%) sensitivity and specificity of the *NRG1* FISH test were encouraging. Notably, we only encountered a single 3′-*NRG1* signal FISH-positive pattern that was strongly associated with RNAseq-validated *NRG1* fusions in previous studies [18,19,20]. In this manner, in the case of no access to RNAseq, the *NRG1* FISH test appears as a robust method for searching for tumors with *NRG1* fusions. Nevertheless, efforts should continue to implement the gold-standard RNAseq as a first-line test when analyzing PDADK and LDADK samples, particularly because of its advantages over FISH in multiplex analyses and precisely in identifying fusion genes and their partners.

## 5. Conclusions

To conclude, the increasing use of RNAseq analyses in solid cancers will keep on improving access to gene fusion diagnosis such as in *NRG1*. Ancient methods such as *NRG1* FISH testing are also valuable due to their excellent sensitivity and specificity (1) in the case of no access to RNAseq or (2) non contributive RNAseq analyses due to the insufficient quality and/or quantity of tumor-extracted RNA.

## Figures and Tables

**Table 1:** Summary of the main features of the two cases series.

Pancreatic Ductal Adenocarcinomas	*n* = 199
Men	112 (56.3%)
Women	87 (43.7%)
Ages	mean 68.4 years (range from 36 to 87 years)
Pancreatic biopsies	95 (47.7%)
Pancreatic surgical specimens	104 (52.3%)
**Lung Adenocarcinomas**	***n* = 446**
Men	251 (56.3%)
Women	195 (43.7%)
Ages	mean 68.4 years (range from 36 to 87 years)
Lung samples	304 (68.2%)
Nodal metastases	39 (8.7%)
Other distant metastases	103 (23.1%)

**Table 2:** Summary of clinical, pathological, and molecular data of the cases with *NRG1* fusions.

Cases	Sex (M/F)	Age Range (Years)	Smoking Habit	Clinical Stage *	Tumor Type and Immunohistochemistry Results	*NRG1* FISH Result **	*NRG1* RNA Seq Result
Lung #1	F	82	No	IVB	Lung solid ADK TTF-1−	50% single 3′	NA
Lung #2	M	78	Yes	IIA	Lung invasive mucinous ADK TTF-1+	70% single 3′	NA
Lung #3	M	71	Yes	IB	Lung acinar ADK TTF-1+	70% single 3′	NA
Lung #4	M	62	NA	IVB	Lung acinar ADK TTF-1−	80% single 3′	NA
Lung #5	M	85	Yes	IVB	Lung acinar ADK TTF-1−	60% single 3′	*CD74-NRG1*
Lung #6	F	44	No	IIIA	Lung acinar ADK TTF-1+	50% single 3′	*ADAM9-NRG1*
Lung #7	M	53	No	IVB	Lung papillary ADK	80% single 3′	*CD74-NRG1*
Pancreas #1	F	40	NA	IV	Ductal pancreatic ADK TTF1−, CK7+,CK20+, CK19+, Bcl10−	80% single 3′	*ATP1B1-NRG1*
Pancreas #2	M	50	NA	IV	Ductal pancreatic ADK TTF1−, CK7+,CK20+	60% single 3′	*CDH1-NRG1*

M: male; F: female; * staging according to UICC TNM classification 8th edition; ** no split and no single 5′ signals were observed; FISH: fluorescent in situ hybridization; RNA seq: RNA sequencing; ADK: adenocarcinoma; NA: not available.

## Data Availability

Detailled data is unavailable due to privacy or ethical restrictions.
